# Reclaiming legacy and envisioning futures in STEM education: Afrofuturism and a scholar-activist-community coalition

**DOI:** 10.3389/fsoc.2026.1907915

**Published:** 2026-10-14

**Authors:** Michele R. Williams, Isaiah M. Stewart, A. L. Rouse, Jayden Seay, Lucienne J. Petit, Melvin Woolfolk, Donovan L. Flagg, Kobe Jordan, Immanuel Stewart, Terrell R. Morton

**Affiliations:** 1 Department of Educational Psychology, University of Illinois Chicago, Chicago, IL, United States; 2 Department of Educational Policy Studies, University of Illinois Chicago, Chicago, IL, United States; 3 Department of Physics, University of Illinois Chicago, Chicago, IL, United States; 4 Department of Mechanical and Industrial Engineering, University of Illinois Chicago, Chicago, IL, United States; 5 School of Public Health, University of Illinois Chicago, Chicago, IL, United States

**Keywords:** Afrofuturism, biology, culturally relevant pedagogy, gatebreaking, post-secondary STEM

## Abstract

**Introduction:**

Amidst growing efforts to dismantle diversity, equity, and inclusion (DEI) policies and programs, educational ecosystems continue to position Science, Technology, Engineering, and Mathematics (STEM) as meritocratic institutions. This disposition is proven inaccurate in higher education STEM culture, perpetuating the marginalization of Black people in STEM. In an effort to enhance Black students’ STEM educational experiences and outcomes, the Re-Envisioning Culture Network (REC-Network) established a coalition of professors, K–12 educators, artists, community partners, and activists to reimagine undergraduate biology curriculum through an Afrofuturistic lens. However, in 2025, funding for the REC Network was subject to the enforcement of new United States federal guidelines that now characterize human-centered research promoting DEI as a violation of anti-discrimination laws, resulting in the immediate cancellation of more than $1 billion in grant funds.

**Methods:**

This critical qualitative case study of the REC Network unearths its strategizing and collective actions to transform dominant STEM power structures in ways that honor historical Black STEM narratives while engaging in liberatory imaginations. Data sources were gathered from four participant groups tasked with redesigning curriculum content and providing supplemental materials. Routine meetings were conducted among authors to deductively code lesson plans, identify emergent findings, and produce final themes from the data.

**Results:**

Three themes emerged from the analysis. The theme of Reckoning with Black Marginalization illuminates the systemic process by which Black individuals and their community are persistently othered by those who identify as members of the dominant racial group in U.S. American society. The theme of Reclaiming Black Legacies situates the components of Black life often lost across the dimensions of time, place, and space. In our analysis of the biology lessons, Afrofuturist principles consistently create opportunities to foster space for speculative imagination and freedom-dreaming, leveraging imagination as a productive cognitive stance rather than mere distraction; this imaginative work invited students to conceive beyond present constraints and to use storytelling and future-oriented scenarios as tools for Re-envisioning Black Possibilities.

**Discussion:**

Our findings demonstrate how a scholar-activist-community coalition situated within a race-critical futurity framing can “gate-break” the prevalent, exclusionary Western and Eurocentric scientific onto-epistemologies, even among contentious geo-sociopolitical contexts.

## Introduction

Despite increases in Black student enrollment in four-year institutions and their expressed interest in studying the biological sciences, the percentage of biology degrees awarded to Black students from four-year institutions has remained disproportionately low for the past 25 years (Riegle-Crumb et al., 2019). Recent reports show that approximately 8%–8.8% of all four-year degrees awarded in the biological sciences from 2000 to 2024 have gone to Black students, despite 20%–25% of Black students entering four-year universities expressing interest in majoring in the biological sciences or science, technology, engineering, or mathematics (STEM) degrees (National Academies of Sciences, Engineering, and Medicine, 2023; U.S. National Science Foundation, 2026). Retaining Black students in the biological sciences is challenging when systemic barriers constrain access to educational opportunities, and science curricula provided to all students do not reflect Black students’ realities, needs, and outcomes (Morton et al., 2023; Gholson and Wilkes, 2017). Morton (2023) notes that among many gatekeeping practices in STEM is the intentional exclusion of Black perspectives and experiences in STEM curriculum.

An approach to supporting Black students’ retention in biology is to radically redesign the curriculum (Morton, 2023). Rather than upholding the gatekeeping traditions of STEM curriculum that foster hostile, toxic learning spaces for Black students, radically redesigning STEM curriculum can serve as a “Gatebreaking” opportunity, as scholars note the structural transformations that can occur through this endeavor across the STEM ecosystem (Brown and Morton, 2023, Miles et al., 2025; Holly and Quigley, 2022). Gatebreaking constitutes the intentional disruption of oppressive systems and structural barriers that prevent Black students from fully accessing and engaging in STEM spaces as their whole, authentic selves, while also promoting their holistic well-being. Gatebreaking can thus occur in myriad ways, including re-envisioning curriculum, pedagogy, classroom management practices, institutional policies and procedures, and cultural norms. For example, in discussing opportunities for Black girls in STEM, Williams et al., (2026) note gatebreaking opportunities that can occur through curriculum as they demonstrate the possibilities of incorporating cultural and historical knowledge into STEM curricula as a strategy to signal to Black girls that they belong in STEM and that their onto-epistemologies—the integration of knowing, doing, and being—are reflected in STEM. Our work extends this thinking to include Black students at the post-secondary level, as we purposefully layered activities and assessments into biology lessons that drew on the Black community’s connections and STEM knowledge. As such, in the context of this study, gatebreaking refers to intentional strategies utilized to re-envision the biology curriculum and account for historical and cultural Black ontologies and STEM innovations, actively dismantling gatekeeping curriculum structures that have excluded Black ways of knowing, doing, and being in biology.

One approach to curriculum redesign is to use a critical, strengths-based framework to guide the design and implementation of all activities, assignments, and classroom management practices associated with the curriculum. As such, this study explores the possibilities of STEM gatebreaking by uplifting the work of the Re-Envisioning Culture (REC) Network, a group dedicated to reimagining introductory biology curricula through an Afrofuturist framework, fostering an inclusive environment that supports Black biology students as an alternative organizational network. Afrofuturism centers Black culture and experiences while reimagining the future through speculative storytelling, science fiction, liberation, and non-Western conceptions of time (Toliver, 2026; Womack, 2013). As a conceptual framework, Afrofuturism guided our lesson planning design by highlighting Black contributions to science and centering its principles as gathered from the literature.

The following research question guided our study:

1. How were Afrofuturist principles utilized by the REC Network to reclaim Black knowledge systems and stories in undergraduate biology curricula?

In the following sections, we outline relevant literature on STEM culture, Black STEM Experiences, and Culturally Relevant Pedagogy. After that, we introduce Afrofuturism as the conceptual framework, establishing it as a tool for reimagining biology education. We outline our methodology as an intrinsic case study to document the Re-Envisioning Culture Network’s collective, interdisciplinary approach to developing an Afrofuturism-inspired post-secondary biology curriculum. Next, we present our findings, organized into three themes: *Reckoning with Black Marginalization, Reclaiming Black Legacies, and Re-envisioning Black Possibilities*. The discussion examines how our themes employ gatebreaking methods as a more inclusive approach to teaching biology. Finally, we conclude by outlining considerations for future research to advance the transformation of STEM education for Black students in the biological sciences.

## Literature review

The bodies of literature that informed this study fall into three categories: STEM culture, STEM experiences, and culturally relevant pedagogy. The literature reviewed discusses the experiences of Black undergraduate students and their perceptions of their STEM disciplines.

### STEM culture

Researchers define STEM culture as being socially constructed to center the white, middle-class male identity and associated ideology as the standard or norm (Morton and Parsons, 2018; King and Pringle, 2019; Mensah and Jackson, 2018; Pinder and Blackwell, 2014). Culture, in this context, is defined as the disciplinary norms, values, beliefs, and practices (Parsons and Carlone, 2013), where STEM learning environments foreground meritocracy and foster competition among STEM students (Martin, 2013). Carlone and Johnson (2007) report that STEM culture privileges Western ideologies by excluding non-Western ways of understanding STEM, acknowledging Eurocentric curricula and pedagogical praxis, and reinforcing deficit stereotypes for those who fall outside these dominant cultural identities. These ideologies manifest in course content (e.g., what is taught and why), norms (e.g., which ideas are presented as universal and why), and practices (e.g., how information is presented and how students are meant to engage in STEM learning) that students experience (Morton, 2023; Barsoum et al., 2024).

For example, pedagogically, presenting problems of focus and solutions in STEM from a hegemonic, normative viewpoint assumes that all people experience and respond to the same phenomenon in exactly the same way, ignoring how various identities influence their science learning experiences (Esparza et al., 2024). This includes introductory biology courses and learning spaces that biology education researchers note as prime sites for engaging with racism, race, and racial equity content (Scheuermann et al., 2024). Many STEM faculty, however, struggle to engage in this practice, as they often maintain and perpetuate a color-blind ideology in their classroom practices (Russo-Tait, 2022) and tend to avoid conversations about race or to not acknowledge anti-Black actions occurring in classroom settings (King et al., 2022). At the institutional level, “weed-out” courses and instructors perpetuate negative stereotypes towards Black students and insult their STEM intellectual abilities (Roby et al., 2022; McGee, 2020; Mutegi, 2013). Weston et al. (2019) define the term “weed-out” course as a subset of introductory, foundational courses students typically take in the first or second years of college. STEM instructors consistently encourage Black students to change their majors or not enroll in STEM courses altogether because STEM professors hold deficit-oriented stereotypes about Black students, such as that they are lazy, uninterested, or incapable of learning STEM (Wilkins-Yel et al., 2019; Marshall et al., 2021). Consequently, undergraduate STEM learning environments continue to emphasize individual accomplishments and fail to meaningfully account for Black students’ ways of knowing, being, and understanding the world, ultimately signaling to Black students that they do not belong in STEM.

Furthermore, the impact of being Black in biology, compared to other STEM disciplines, requires examining how this broader culture of exclusion within STEM manifests specifically in biology. There are key differences across the STEM fields (Reinholz et al., 2019). Biology curricula tend to have less rigid course sequencing than in fields like mathematics, where students can often test into higher-level courses. Additionally, unlike mathematics or physics, biology is not believed to require innate knowledge or talent. In contrast, success in fields like mathematics and physics is often attributed to natural ability shaped by gender and age. Biology instruction places more emphasis on memorization than on application, unlike the computational procedures in mathematics. Also, biology is more gender-balanced, with more equal representation of women and men, while physics has been described as predominantly white and male (Reinholz et al., 2019). Taken together, these characteristics contribute to the perception that biology is an easier or less rigorous STEM field.

### Black STEM experience

Black onto-epistemologies are plural, dynamic, and continually shaped by a multitude of experiences, cultural traditions, geographic locations, identities, and perspectives within communities and across the diaspora (Morton, 2026; McKittrick and Woods, 2007; Ortiz, 2020). Likewise, Black students’ STEM experiences should not be understood as uniform but as shaped by their intersecting identities (e.g., gender, class, nationality, and (dis)ability) and their educational background (e.g., major, institution type). These factors influence how they engage with science learning and envision Black futures (Morton and Parsons, 2018; Berry et al., 2011; McGee and Martin, 2011).

For example, research focused on Black women in STEM notes the gendered racism experienced by Black women compared to Black men and white women (McGee and Bentley, 2017; Ong et al., 2011). This body of work notes how gender intersecting with race creates a unique experience that is multiplicative rather than additive, where in racially progressive STEM spaces, Black women can be othered given the absence of a gendered consideration (e.g., Morton, 2021). Gendered-racism considerations have also been extended to studies of Black men in STEM, noting the specific ways in which interlocking systems of oppression operate on them, given stereotypes of hyper and toxic masculinity (White and Morton, 2024). Likewise, Black Queer students face invisibility via two means: cultural erasure and within-group peer tensions (Leyva et al., 2022), where Black Queer STEM students feel unmotivated and unwelcome in environments that do not attend to the intersections of race, gender identity expression, and sexuality. This includes spaces and curricula designed to be race and gender-inclusive, in which gender is presented as binary (Leyva et al., 2022). Examining the intersection of race and disability is another example of how institutional policies and practices can facilitate different experiences and outcomes for students with disabilities, both neurological and physical, as they are often not considered through an intersecting lens that is racialized (Annamma et al., 2013).

Across a multitude of experiences, a recurring theme emerges in the literature: most Black students describe their undergraduate STEM environments as unwelcoming, with this association tied to institutional, disciplinary, and programmatic structures (Basile and Black, 2019). Across disciplines, campuses, and regions, students recount the many ways their identities, histories, and intellectual contributions are dismissed or outright invalidated (White and Morton, 2024; McGee and Martin, 2011). These patterns surface in a range of institutional settings, from predominantly white institutions (PWIs) to Hispanic-serving institutions (HSIs) and other Minority Serving Institutions (MSIs) (Flores et al., 2023; Wells-Stone et al., 2026).

In response, scholars continue to stress the value of learning spaces that celebrate and embolden Black learners. Black STEM students can, in fact, thrive at PWIs and MSIs when systems are built deliberately to support their cultural identities and ontologies (Boyd-Sinkler et al., 2024; Ellis et al., 2026). Historically Black Colleges and Universities (HBCUs) have generally done a better job of supporting Black students holistically (Morton, 2021; Ellis et al., 2026). Even so, these comparatively supportive campuses are not immune. Discrimination can still radiate from their STEM departments, where the steady hiring of predominantly White and Asian faculty perpetuates negative stereotypes about Black STEM learners (McGee, 2016). Students at institutions supposedly designed to affirm Black identities have reported feeling that their intellectual abilities were on trial even after passing their STEM courses, an experience echoing that of Black STEM students elsewhere (McGee and Martin, 2011). Rodgers (2024) presses further, examining what happens to Black women when the gender and racial composition of faculty fail to reflect the students they are meant to support. Black learning spaces are invaluable, and they measurably improve students’ chances of success. As practitioners work to strengthen these spaces, they must be willing to rebuild these educational environments as more collectively connected, gender-affirming, and culturally relevant.

### Culturally relevant pedagogy

As barriers for biology education at the post-secondary level are uncovered, there are opportunities to address them through culturally relevant pedagogy (CRP). As defined by Gloria Ladson-Billings (1995), CRP is a framework that promotes academic excellence and cultural competence and fosters critical consciousness to challenge systemic inequities. Academic success comprises holding all students to high expectations while ensuring they do not fail as they engage in rigorous academic content, where cultural competence is seen as connecting students’ cultures, communities, and lived experiences to the curriculum as an entry point to new learning. Critical consciousness involves creating opportunities for students to identify and critique social inequities in their communities and devise methods to address them (Ladson-Billings, 1995). CRP has been promoted in STEM education for its ability to help foster inclusive STEM learning environments (Williams, 2026; Edwards and King, 2023). Core to its principles, though often the most left out in science teaching, is CRP’s emphasis on fostering critical consciousness, or sociopolitical consciousness (Madkins and McKinney de Royston, 2019). Madkins and McKinney de Royston (2019) note the importance of the educator maintaining political clarity in efforts to foster sociopolitical consciousness in science teaching; a practice that can push science educators to grapple with racism as fundamental to science curriculum design and science teaching (Sheth, 2019). Contemporaries of CRP include Hip-Hop pedagogy (Ladson-Billings, 2014) and Culturally Sustaining Pedagogy (Paris and Alim, 2014). Culturally Sustaining Pedagogy (CSP) is understood as honoring and valuing cultural practices of students with marginalized backgrounds while simultaneously offering space to critique oppressive structures (e.g., racism and misogyny) that persist in today’s society. It was presented as “loving critique forward,” in which Paris and Alim challenged educators to move beyond making something relevant to sustaining the practices in ways that make a sustained impact on student outcomes. Paris and Alim (2014) also reorient conversations back towards a more multicultural approach with CSP; however, some scholars note that such perspectives promote anti-Blackness and marginalize Black people, particularly when considering how Black language is taken up and implemented in classroom spaces (Ortiz and Ruwe, 2021).

In the context of biology education, CRP is a powerful tool for addressing the historical marginalization of non-dominant cultural epistemologies and perspectives. The dominance of Eurocentric narratives in science curricula continues to alienate many students, particularly Black learners, from the discipline (Bala, 2006; Biraimah et al., 2024). By grounding biology instruction in culturally relevant practices, educators can dismantle systemic barriers and reframe biology as inclusive and transformative (Mensah, 2011; Marosi et al., 2021). Despite growing acknowledgment of these inequities, biology education still largely lacks pedagogies that center students’ cultural identities and lived experiences (Morton, 2023).

Afrofuturism envisions Black futures through narrative reclamation and reimagined science, offering a compelling addition to culturally relevant pedagogy (Gipson, 2019). Gipson (2019) and White et al. (2026) illustrate how Afrofuturism can reimagine STEM spaces as culturally affirming and imaginative, enabling students to see themselves as scientists and creators of new futures. Integrating Afrofuturism into biology education not only disrupts Eurocentric science but also provides students with tools to question, critique, and reshape scientific paradigms (Anderson, 2016). However, the literature remains limited in its exploration of the operationalization of Afrofuturism within science curricula, particularly in biology, and its potential as a transformative pedagogical strategy. The following section of our study addresses this gap by examining how Afrofuturism can support culturally relevant pedagogy in undergraduate biology education.

## Afrofuturism

Afrofuturism, as a conceptual framework, critiques the present, (re)envisions the past, and (re)imagines societal institutions through a Black cultural lens for the future (Holbert et al., 2020; McGee and White, 2021; Womack, 2013). Afrofuturism’s fundamental symbol, the Sankofa bird, is an outward signifier of “looking back to go forward,” representing the future by remembering the past (Strait and Conwill, 2023). Afrofuturism thus oscillates between ancient histories and present-day society to illuminate and understand Black peoples’ lived experiences, both historically and in the present (Strait and Conwill, 2023). Furthermore, its conceptual connections to Yoruba, Bokongo, and Dongon cultures allow the framework to resist Western forms of racism, legacies of enslavement, and their subsequent effects on the Black community, including the erasure of our collective Black memories from history textbooks. As the pendulum swings back, it offers space to (re)create possibilities in which Black people are seen in futures that might be unimaginable to most people. Conceptually and pragmatically, Afrofuturism existed among Black authors, musicians, artists, and cultural movements more than a century before Mark Dery coined the term Afrofuturism in *Black to the Future*. Dery’s (1994) definition of Afrofuturism suggests that:

Speculative fiction that treats African American themes and addresses African American concerns in the context of twentieth-century technoculture – and, more generally, African-American signification that appropriates images of technology and a prosthetically enhanced future – might, for want of a better term, be called ‘Afrofuturism.’ (p.180).

As a result, Afrofuturism is seen in literature and scholarship and broadly influences African-American culture, music, art, fashion, and film. Scholars theorize Afrofuturism as a method for critiquing Black girls’ current social context and how they endure racism or gendered oppression, while simultaneously reimagining and illuminating possible futures for Black girls (Holbert et al., 2020; McGee and White, 2021). Previous researchers have fused constructionism and Afrofuturist methodologies, which encompass connecting with the past by embedding personal and family histories, reflecting on the present to contest systemic inequities in the community, and looking to the future to center Black voices, experiences, perceptions, and values (Holbert et al., 2020). Similarly, Strait and Conwill (2023) state that Afrofuturism, as a framework, includes an interpretation of the history of race and its nuanced experiences connected to Black cultural identities. Afrofuturist methodologies provide a framework to interpret race and the consequences the Black community endures as we navigate society’s oppressive and systematic structures that continue to disenfranchise Black people (Strait and Conwill, 2023). Combined, these understandings of Afrofuturism guide research through a historical connection to the African Diaspora, interpreting the present and (re)imagining Black futures.

### Principles of Afrofuturism

Although previous scholars disagree on specific tenets of Afrofuturism, the following are commonalities across several texts. These tenets, as articulated in Williams et al. (2026), include feminine (identity), alienation (otherness), time (cyclical rather than linear), science fiction (space), utopian realities (possibilities), Blackness (cultural diversity/affirmation), and liberation through acts of social justice for the Black community (see Table 1). Table 1 provides specific definitions and an example for each tenet, as understood by the author and colleagues (2026). These themes fall under a broader Sankofa understanding, in which Sankofa refers to looking back to and leveraging ancestral histories to move forward and forge new possible futures (Opare, 2025). Within this manuscript, Afrofuturism serves as a race-critical futurity framing, allowing our scholar-activist-community coalition, REC Network, to interrogate and disrupt Western and Eurocentric scientific onto-epistemologies while working within the controversial geo-sociopolitical context.

**Table 1:** This table highlights principles connected to Afrofuturism as understood by Williams et al., (2026).

Term	Definition	Example
Feminine Identity	A feminine identity focuses on the intersection of being both a woman and Black in society, celebrating the resilience and strength of Black women (Strait and Conwill, 2023; Toliver, 2026; Womack, 2013).	It explores their connection to nature, often symbolized by the idea of Mother Earth, highlighting their ability to nurture and sustain.
Alienation (otherness)	Alienation or otherness describes when Black people are racialized and considered outsiders because of their skin (Strait and Conwill, 2023; Yaszek, 2006).	Examining the social construct of race and the real consequences of discrimination due to social inequities that Black people experience.
Time	Understanding time through an Afrofuturist lens does not assume that a Western understanding is linear and unidirectional (Strait and Conwill, 2023; Womack, 2013). Instead, Afrofuturists cyclically utilize time, always able to return and learn from the past, which informs the present.	Afrofuturists consider alternative realities where the past might differ from what we know today, and emphasize that our futures are not fixed because of our current circumstances.
Futurity & Worldbuilding	Futurity & Worldbuilding are central metaphors that combine to encourage Black people to dream about new possibilities, futures, solutions, and worlds (Strait and Conwill, 2023; Toliver, 2026; Womack, 2013; Yaszek, 2006).	Not just the cosmos, but also the physical and mental space to escape their current realities and use their imagination to build worlds where Black people can author themselves in spaces.
Liberation	Finally, liberation offers a sanctuary that frees Black people from the strict and unilateral understanding of time, space, and reality (Strait and Conwill, 2023; Womack, 2013; Yaszek, 2006).	It creates a space where the notions of hope, reclamation, and joy are experienced in a future free from societal constraints.
Blackness	Blackness is defined as the outward expression of the racial, ethnic, and cultural identities of people of African ancestry. It is performed in multiple ways while being connected to a Black person’s societal position of being ‘othered’ due to experiencing multiple oppressions created by the dominant racial group, which impacts how Black people are perceived and treated in American society (hooks, 2015; Mills, 2020; Morton et al., 2019; Womack, 2013).	Through the Black communities’ expression of individuality and ethnicity, Blackness can include outward performances such as music, hairstyle, clothes, and even the language spoken to demonstrate how Black people are different from one another, even as the dominant racial group in American society has categorized all Black people as the same.

Williams et al. (2026).

## Methods

This research study is an intrinsic case study of the REC Network, exploring how this group came together to develop Afrofuturist biology lessons. As an organizational coalition, the REC Network signified the bounded case. Primary and secondary data sources consisted of lesson plans, pedagogical resources, and assessment materials. They were collected during meetings, workshops, and think tanks (Miles et al., 2020).

### Positionality

Our research team is interdisciplinary, and as such, our collective positioning as Black scholars, researchers, educators, dreamers, activists, and students reflects our lived experiences, intersectional identities, and multidimensional bodies of knowledge. We share an investment in justice-oriented STEM education research. As such, approximately 60% of the authors are positioned as insiders, who have navigated STEM environments as Black undergraduates, with the remaining authors identifying as co-conspirators in the movement to dismantle oppressive structures and reimagine STEM education toward Justice and Joy. As a collective, our research interests intersect through a shared passion for educational research and scholarship to reimagine STEM education and empower under-resourced communities through culturally relevant frameworks (Ladson-Billings, 1995). As members of the REC Network, our critical-constructivist orientation guided our research design to analyze how adaptations of Afrofuturist content contribute to the transformation of dominant cultures in undergraduate biology education (Morton, 2023; Denzin, 2015; Holbert et al., 2020). Our incorporation of Afrofuturism as an interdisciplinary vehicle for rigorous, analytical scholarship to reckon with the past, interrogate present systemic and oppressive structures, and radically imagine futures for Black STEM identities marks the disciplinary transcendence of our communal efforts.

### Research context

The REC Network is a multigenerational, interdisciplinary collective of forty Black biology faculty, scholars, researchers, professionals, community activists, Black cultural experts, and artists. The members of our community come from a variety of institutions, including Historically Black Colleges and Universities (HBCUs), Hispanic-Serving Institutions (HSIs), Predominantly White Institutions (PWIs), Research One institutions (R1), liberal arts colleges, teaching institutions, secondary schools, and community organizations. The REC Network organized in-person think-tank sessions where, as a community, we engaged in freedom-dreaming activities, envisioning introductory biology without systemic barriers. Data collection spanned approximately 4 years. This included hosting guest speakers who incorporated Afrofuturism in their research, creating a repository of historical figures in STEM to integrate into lessons (to learn from the past), and utilizing technology resources that provide students with the creative space and freedom to express their learning.

The initial Fall 2020 virtual think tank laid the groundwork for understanding the current state of undergraduate biology education, focusing on the cultural norms, values, beliefs, ideologies, and practices that shape Black students’ experiences. This think tank was followed by small-group meetings in Spring 2021, Fall 2021, and Spring 2022. These sessions focused on refining the network’s aspirations, deepening the members’ understandings of Afrofuturism, and readily identifying anti-Black systems in biology education. In Fall 2022 and Spring 2023, the network published a call to action (Morton et al., 2023). In Fall 2023, a hybrid think tank at North Carolina Agricultural & Technical State University brought participants together to begin creating Afrofuturist-inspired biology lessons. Through spark talks, group sessions, and collaborative discussions guided by our questions (see Appendix A), participants explored biology topics such as mitosis, DNA, and cancer through Afrofuturist perspectives, integrating storytelling and cultural relevance.

In Spring 2024, an additional virtual workshop supported the continued development of these lessons. Four collaborative groups proposed guiding questions, drafted sample lessons using our Tips for Developing Afrofuturistic-Oriented Instruction Activities (see Appendix B), and discussed the intersections between Afrofuturism and course-based undergraduate research experiences (CUREs). There were four collaborative groups working on creating Afrofuturism-inspired biology lessons. In the Fall of 2024, the REC Network partnered with the International Conference on Urban Education (ICUE) and conducted a preconference session where members of the REC Network convened to finalize lesson plans, refining lesson instructional materials such as learning tasks for student engagement, instructional slide decks for biology instructors, and assessments using our Tips for Developing Afrofuturistic-Oriented Assessments (see Appendix C) to gauge student learning and mastery of biology concepts and skills.

### Data sources

Textual and audiovisual artifacts were collected and transcribed from meetings, workshops, and think tanks that occurred between 2020 and 2025. These artifacts were developed by working groups tasked with redesigning undergraduate biology curricula utilizing principles of Afrofuturism, culminating in lesson plans and supplemental instructional resources. Each of the four working groups was facilitated by a researcher who also observed the dynamics of collaboration. The groups each completed one lesson plan, integrating foundational biological concepts such as mitosis, cancer, and DNA with principles of Afrofuturism, which were used as the primary basis for coding. Participants were assigned to working groups to foster a collaborative approach to enhance traditional biology concepts by incorporating Afrofuturist adaptation templates. These templates, referenced as *Appendices A, B,* and *C*, served as pedagogical guides to help participants collectively organize and scaffold the development of their reimagined lesson plans and instructional resources.

Four groups, each with at least five participants, were intentionally situated based on areas of expertise. For example, this included pairing together professors, secondary science teachers, and artists to enrich the design process. This iterative structure enabled the repeated engagement necessary to establish a shared understanding of what it means to incorporate Afrofuturism while immersed in the redesign of undergraduate biology curricula. The final dataset consisted of an Excel repository containing 40 articles, websites, instructional slide decks, lesson plans, infographics, summative and formative assessments, and pedagogical templates.

### Data analysis

Our methods of data analysis consisted of four authors separately coding educational curricula in accordance with the codebook, followed by weekly review meetings in which authors discussed points of clarification, identified emergent themes, and resolved disagreements after presenting analytic justifications to the team. The team constructed a codebook including six Afrofuturist principles drawn from the literature: Alienation, Blackness, Feminine Identity, Liberation, Time, Futurity/Worldbuilding, which allowed for the categorization of data according to the above principles. Each author developed an individual analysis of a pre-assigned dataset (e.g., Lesson Plan 1 materials) while making iterative revisions organized in a master spreadsheet that contained the codebook, a complete list of codes, and the analyzed outcomes. Meeting cadence involved providing space to express any conflicts experienced during the engagement with coding methods, anchoring the group in codebook definitions when moments of conflict emerged (e.g., confusion, disagreement, personal reflection), and providing time to reflect on findings or progress from the previous week. Deductive coding was the primary method for analyzing the qualitative data. Saldaña (2021) describes deductive coding as “theory-driven,” making it appropriate for this study, which began with established principles from Afrofuturism to examine their manifestation in curriculum design. Coded excerpts were grouped by themes, with contextual information indicating the lesson from which each excerpt came. This allowed us to identify and review patterns across all lessons. The data were housed in an Excel spreadsheet, which served as a central location for analyzing raw data and interpreting them, providing a transparent and traceable path from evidence to thematic insights (Nowell et al., 2017). Coding with this theoretical lens allowed us to uncover codes of historical misrepresentation, communal empowerment, and acts of futuring that emerged throughout biology learning goals, lecture materials, and lesson activities (Zhang and Wildemuth, 2009).

All codes were gathered from the codebook housed within the Excel spreadsheet. Coded data and codes were organized according to Afrofuturist principles to identify emerging themes. Emerging themes were routinely revisited and critically analyzed to assess the following:

1. Prevalence of code associations across lesson plans.
2. Depth of the supporting evidence (i.e., associated data excerpt, code, and corresponding memo provided for justification).

Emergent preliminary themes were recalibrated during research team meetings as an ongoing practice to “…assemble chunks of data that go together, and to further condense the bulk into readily analyzable units.” (Miles, Huberman, and Saldaña, pp. 64, 2020). Codes that intersected across categories were organized to correspond to the final three themes: *Reckoning with Black Marginalization*, *Reclaiming Black Legacies*, and *Re-envisioning Black Possibilities*. The research team ensured that emerging themes in the data aligned with the tenets of Afrofuturism by associating key terms and excerpts with multiple codes, as relevant (see Figure 1).

**Figure 1:**
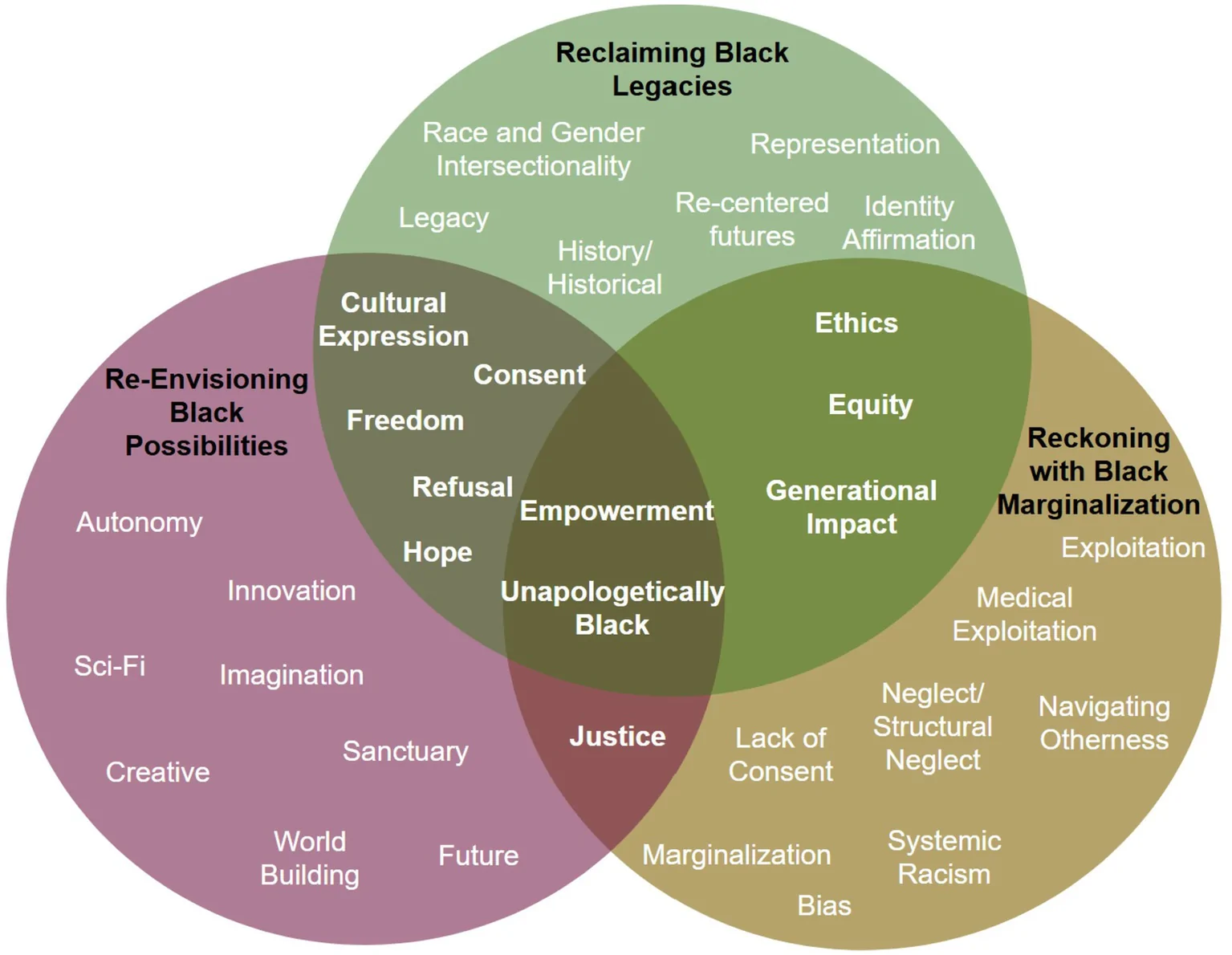
Thematic analysis codes aligned to findings’ final themes: codes applied during the deductive coding process, along with corresponding data exemplars, were used to derive the final themes for the findings. The codes were then organized using a Venn diagram to illustrate the interconnectedness between our themes. In this figure, black text reflects the theme names: Green Circle–Reclaiming Black Legacies; Red Circle–Re-envisioning Black Possibilities; Yellow Circle–Reckoning with Black Marginalization. White text represents the codes used throughout the coding process. Codes grouped in overlapping spaces between circles, with bolded white text, indicate their intersection points across themes.

For instance, instructional materials that invited students to transform sites of historical environmental racism that created food deserts were conducted by mapping access to fresh produce. This activity empowered students to familiarize themselves with alternative pathways to healthy food, community spaces, and sites of love. This radical act of *Re-envisioning Black Possibilities* by imagining equitable futures was coded under the tenet of Liberation in Table 1 identified by the codes of imagination, world-building, and innovation, which ultimately manifested in contexts of localized autonomy through the centering of sustainable food ecosystems (Lesson Plan 3, Learning Tasks). Table 2 provides a visual representation of the coding process replicated across all instructional curricula to ensure consistency with the conceptual meanings of each theme.

**Table 2:** This table illustrates an exemplar piece of data and its categorization using codes.

Curriculum exemplar (data source)	Afrofuturist principle	Initial codes	Analytical interpretation	Final theme
Mapping access to fresh produce (Lesson Plan 3, Instructional Slide Deck (13) – Websites)	Liberation	Recentered futures, sanctuary, imagination	Empowering students to familiarize themselves with alternative pathways to healthy food, spaces for community, and sites of love.	Re-envisioning Black Possibilities

It also includes the data source from which this data can be referenced and a justification for why it falls under its code and final theme.

## Findings

Our findings demonstrate our conceptualization of the process in radically redesigning biology curricula to implement culturally responsive STEM education, which built opportunities to dream of possible liberated Black futures. By applying Afrofuturist principles, we highlight the intentional Gatebreaking curriculum decisions made by the REC-Network that identify discrimination against the Black community within the healthcare system, foreground ancestral knowledge, and encourage future possibilities through world-building imagination.

### Reckoning with Black marginalization

The theme of *Reckoning with Black Marginalization* illuminates the systemic process by which Black individuals and their community are persistently othered by those who identify as members of the dominant racial group in U.S. American society. This theme foregrounds the structural inequities in healthcare access and treatment that operate as chronic stress within Black families and communities. The intersection of race and health outcomes is not accidental, but instead the consequence of systemic failures that have shaped how the healthcare institution engages with Black bodies, and it is essential to address the deep entanglement between race and health. As shown in Lesson Plan 2’s instructional slide (see Figure 2), the formative assessment asks the students to explore their own cancer-causing exposures or risks by telling their own stories or a story of someone they know. This particular task connects concepts that students are actively learning about to their personal lives, which creates the opportunity to explore societal, environmental, occupational, and genetic factors that Black people are more vulnerable to, due to the history of systemic racism that produces stress and poverty.

**Figure 2:**
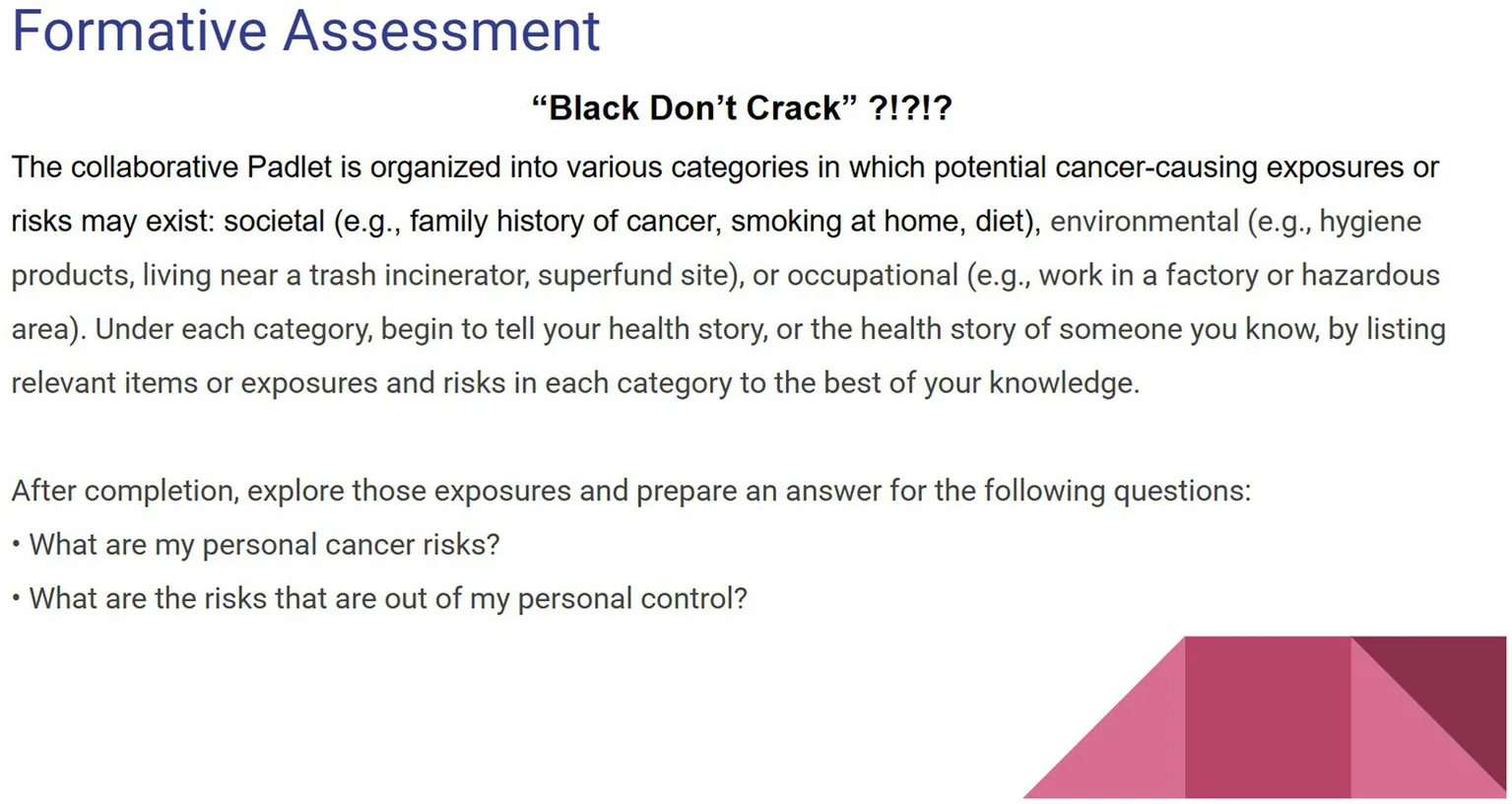
This is an instructional slide from Lesson Plan 2 that demonstrates a formative assessment for a professor to incorporate into their biology course.

Additionally, as articulated in Lesson Plan 4’s Acquisition, “students will be able to explain how cancer impacts the Black community differently, considering factors like unethical research practices and potential environmental exposure”. The goal of this particular lesson is to allow students to examine the impact of cancer within the Black community and reflect on the broader awareness of how systemic racism operates through medicine and the environment. This lesson plan raises awareness of inequities in health care policies and invites reflection on their impact on the life trajectories of the Black community.

Marginalization, as a second intercomponent of this broader theme, does not exist only in social spaces but also within the body. It is through multiple, layered exposures that biological risk is shaped over time. As stated in lesson plan 2’s instructional slide deck for university professors to teach the topic of Cancer Causes articulated through a reading activity titled “Exposure Affects Biological Risk,” how multiple domains of exposure environmental, occupational, societal (socio-political) can have a compounding impact on marginalized communities (see Figure 3). Another example of marginalization is noted once again in Lesson Plan 4’s supportive text, *U.S. Louisiana’s ‘Cancer Alley’*, which highlights for students the impact of marginalization on an entire community, “Many shared stories of entire communities devastated by cancer, the deaths of family and friends, missed days of work and school due to illness, and children rushed to emergency rooms suffering from asthma attacks” (Human Rights Watch, 2024).

**Figure 3:**
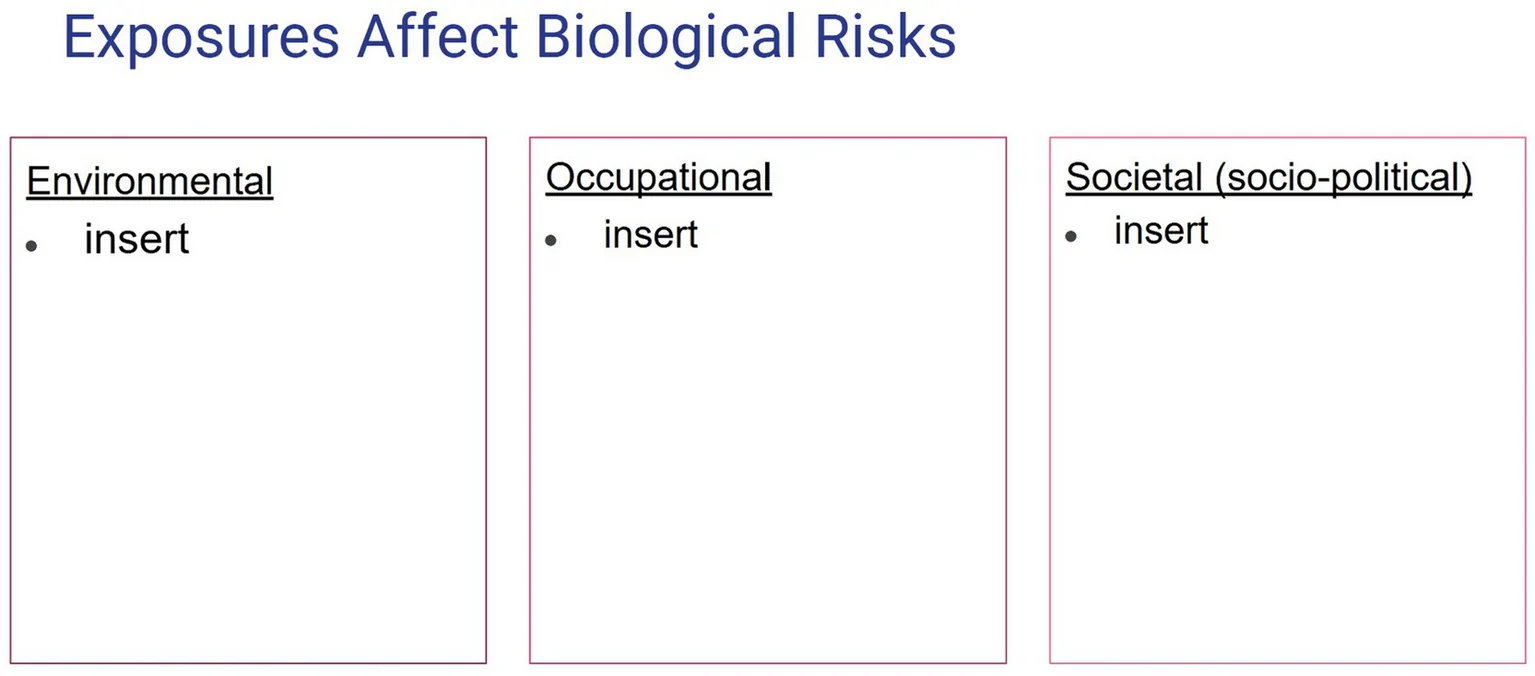
Lesson Plan 2’s instructional slide that demonstrates a learning task for students to engage with the supplemental reading.

A final component of this theme is the lack of consent and medical exploitation that the Black community experiences as they navigate the medical institution. The exploitation of Black bodies within the medical and research institutions has deep historical roots, and its legacy continues to shape distrust and harm within Black communities. Henrietta Lacks and the unauthorized use of her cells offer a notable and consequential example of this type of breach. As documented in lesson plan 4’s supplemental reading, *Henrietta Lacks’ Family’s Lawsuit: Ethical Questions and Solutions*, “Lacks was not told that researchers might receive, use, or share her cells, and for several decades, her descendants did not know that her cells were widely used” (Klitzman, 2022, p. 769). *Reckoning with Black Marginalization* connects the material conditions of marginalized life and how they are treated socially and politically directly to biological vulnerability, underscoring that health disparities are structurally determined instead of randomly distributed.

This reckoning extends well beyond the bounds of our example biology lesson. Black marginalization can be uncovered in topics from evolution to ecology (Bullard and Wright, 1993; Graves, 2001). These extended examinations have the potential to assist students in recognizing both natural and fabricated environments that affect how Black-bodied organisms live and interact with their surroundings (McKittrick, 2020), as was demonstrated by Figure 2 from our biology lesson connected to Cancer Causes.

### Reclaiming Black legacies

The theme of *Reclaiming Black Legacies* situates the components of Black life often lost across the dimensions of time, place, and space. The practice of reclaiming manifests across methods and modes of being that elicit forms of speculative fiction, African genealogical inquiries, and the facilitation of student imaginaries through performance tasks. These restore ancestral stories as sources of power that have been lost, ignored, hidden, and rewritten. This theme demonstrates how restorying and contextualizing Black narratives shifts historical and contemporary sites of injustice into rediscovered sources of empowerment and liberation. Findings depict the intentional centering of Black voices, experiences, and culminating stories in biology, which are normally excluded from traditional science curricula.

Our analysis connected lesson plans with codes of intersectionality, representation, Blackness, time, and science fiction. Intersectional representation is evident in lesson plan 3’s stated goals, “Students will know … Black people who have and are contributing to the study of DNA.” This component is essential because Black scientists and their contributions are systematically erased from contemporary science curricula (Biraimah et al., 2024; McGee and White, 2021; Taylor, 2024).

In lesson 4, Henrietta Lacks provides a powerful example of the historical impact of Black women in science (see Figure 4). The limitless abundance of her cell growth illustrates a nonlinear orientation to Western conceptualizations of time, given the endless supply of an immortal cell line. As a core principle guiding the lesson, lesson plan 1’s essential question poses: “How can one person’s story, like Henrietta Lacks’, transform our understanding and ethical perspectives of science and cells?” This line of questioning prompts students to identify what constitutes informed consent, drawing pathways to the reclamation of bodily autonomy (Nature, 2020).

**Figure 4:**
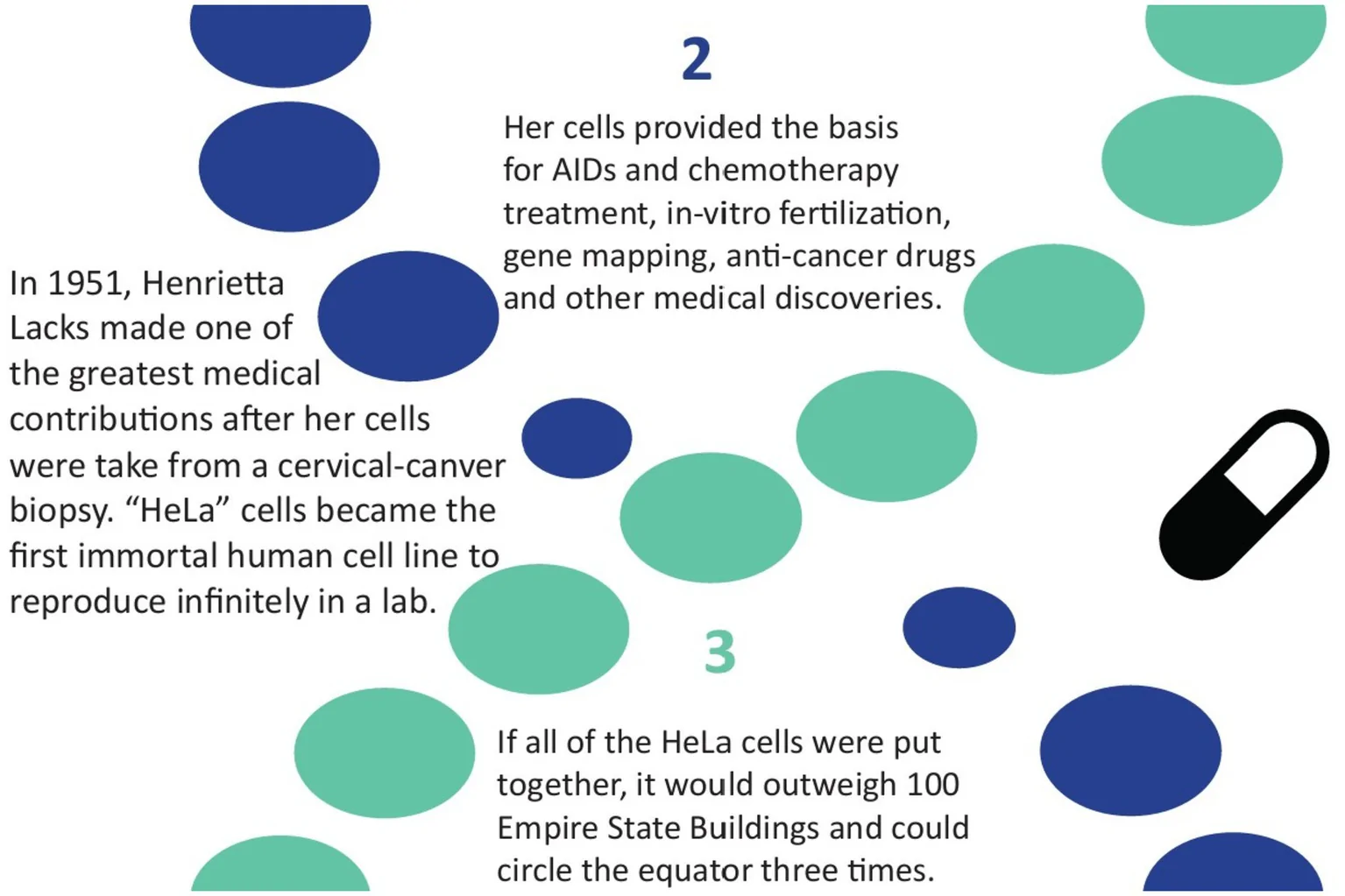
Lesson 4 supplemental infographic resource “5 Henrietta Lacks Facts,” to illustrate her impact in quick, interesting snippets.

Lesson plan 4’s reading displays how Henrietta’s family serves as an example of generational impact, grappling with historical systemic inequities while taking reparative action. In recent years, Henrietta’s profound contributions to science and medicine have become better known. Hundreds of companies were complicit in “exploiting her legacy” (Klitzman, 2022, pp. 769). At the same time, some of Henrietta’s own family members have had to continue navigating poverty and lack basic needs such as health insurance. In 2021, the Lacks’ family started the process of seeking restitution for the nonconsensual use and sharing of Henrietta’s biological property. The family’s lawyers have argued these pharmaceutical and biotech companies need to relinquish net profits to atone for the billions of dollars they have profited from on the back of stolen property (Klitzman, 2022). Weaving Henrietta’s narrative throughout the designed lessons urges students to recognize the generational impact of Black legacies far beyond ancestral bloodlines.

Black legacies unearthed extended to Afrofuturist content adaptations that explored the biological foundation of Black women, deeply intertwined with the overall structure and function of life, while offering the historical and contemporary societal implications of DNA modification. The inclusion of a resource detailing 1,000 current Black geneticists and community scientists positions ancestral knowledge alongside contemporary innovation as reclamatory forces of liberation (Cell Mentor, 2020). Performance tasks, such as lesson 1’s “Build a Black Woman Superhero,” provide students with the opportunity to design legacies of Black futurity through the culmination of science fiction, creativity, innovation, and hope. In their performance assessment of mastering mitosis and reimagining Henrietta Lacks as a Superhero, students are exposed to examples of Black legacies, serving as a guide for pedagogical tools that animate futures grounded in collective-oriented reclamation of Black legacies.

As oppressive narrators have controlled the perceived truth about Black human character and ability in the sciences for over a millennium, we aim to bring students together to familiarize them with Black figures in biology, ensuring that each individual’s life and story are better contextualized (Lund, 2022). This approach has the potential to encourage students to build their own identities in relation to the newly shared narratives and to further interrogate history, motives, and knowledge within the sciences (Wootton, 2015). As such, lesson plan 4’s readings illustrate how the REC Network reclaimed Black legacies by including and contextualizing Black narratives within the biology resources.

### Re-envisioning Black possibilities

In our analysis of the biology lessons, Afrofuturist principles consistently create opportunities to foster space for speculative imagination and freedom-dreaming, leveraging imagination as a productive cognitive stance rather than mere distraction. This imaginative work invited students to conceive beyond present constraints and to use storytelling and future-oriented scenarios as tools for *Re-envisioning Black Possibilities*. In an effort to create a space for student empowerment, a lesson on DNA used genetically modified organisms (GMOs) as an entry point to examine structural inequities in food access. By connecting molecular concepts to the lived realities of food deserts in Black communities, the lesson provided curricula opportunities to encourage students to consider both biological mechanisms and social determinants of health (Reese, 2018). One activity in lesson 3 foregrounded collective agency by asking students to map “access to fresh produce within the students’ community,” leveraging the mapping as a practical strategy for resisting environmental racism. In this way, the lesson transitioned from abstract genetics to actionable, community-centered strategies for local autonomy. Similarly, a lesson on cancer causes invited students to “tell their health story” and “the health story of someone you know” by interviewing elders/community members to document diverse intergenerational health narratives. The incorporation of Black students’ lived experiences into the lesson amplifies voices that are often ignored in traditional science courses, potentially empowering their own STEM identities. Thus, reimagining learning experiences for Black students.

In reimagining Black possibilities, the lessons create curricula opportunities to also engage students’ sense of world-building. Lesson one’s reading highlighted how practitioners and community organizations address conditions such as sickle cell disease by expanding access to care through “providers… traveling to satellite clinics… or attending community outreach events,” illustrating how medical imagination can translate into tangible interventions (UNC Health, 2023). These examples underscore the multiplicity of institutional and grassroots approaches to worldbuilding, taking steps to improve the quality of life for conditions that disproportionately affect Black people. In a push to extend students into a realm of disruption, the designed unit on cancer, “Black Don’t Crack,” asked students to imagine a “world without cancer. In your ‘futuring’ … develop a multi-modal offering that’s reflective of a post-cancer world that acknowledges the history, people, places, and things that are different as a result.” In order for students to visualize the transformation of people, places, and practices, learners must reckon with historical harms (e.g., environmental racism and medical negligence) by broadening the scientific inquiry to include “societal e.g., family history of cancer, smoking at home, diet, environmental (e.g., hygiene products, living near a trash incinerator, superfund site), and occupational (e.g., work in a factory or hazardous area) exposures that can increase an individual’s risk of cancer.” By combining scientific evidence with narrative and cultural reflection, the tasks were designed to encourage students to envision beyond risk factors and statistics, championing collective futures that have not been deemed possible. Across these lessons, acts of *Re-envisioning Black Possibilities* are reflected in Afrofuturist-infused pedagogy that operates as both an intellectual scaffold and as an emancipatory practice. By situating biological content within structural contexts, the curriculum was designed to foster critical reasoning, socio-political awareness, and creative problem-solving among students, thereby legitimizing speculative imagination.

The scale of biology ranges from tiny molecules to the entire biosphere, encompassing all living things in between (Heil et al., 2024). In a time when unconventionality is disparaged, uniqueness is vilified, and creativity is perpetually outsourced, re-envisioning can be more than a simple classroom activity. It can be a powerful inspiration, a guide to propel Black liberation from the theoretical to its infusion at every level of life here on earth (Tynes et al., 2023). Lesson plan 3 was designed to encourage students to combat environmental racism through the fresh food mapping exercise, thus providing them with the tools to re-envision their communities and resources. Taken together, our findings suggest Afrofuturist pedagogy does more than enhance biology curricula and instruction. It creates an environment where Black students can envision themselves as producers rather than just passive consumers of biology knowledge.

## Discussion

This study explored how the REC Network, an alternative organizational coalition, could foster the development of Black STEM identities and enhance science education outcomes by reimagining biology curricula through an Afrofuturist lens. Overall, the following themes illuminate how Afrofuturism can be used to gatebreak access, learning, and engagement in STEM by forming meaning, identity, and utopian possibilities in biology courses. The first theme, *Reckoning with Black Marginalization*, exposes the structural inequities that manifest in content relevant to the biological sciences, like what is seen in healthcare and society, that function as continual issues. *Reclaiming Black Legacies* demonstrates how instructors can recover historically erased knowledge in the teaching and learning of biology. *Re-envisioning Black Possibilities* inspires faculty to embrace the powerful intellectual tools that Black students wield, including imagination, speculative thinking, and storytelling in their learning spaces. Such tools challenge hegemonic discourse about students’ roles and responsibilities in an undergraduate learning environment, namely, the assumptions that students are passive learners or that they cannot grapple with structural racism while studying biology (Chi and Wylie, 2014; Sheth, 2019). Taken together, our findings suggest an Afrofuturist approach to biology curriculum design can do more than enhance instruction. It can create space for Black undergraduate students to engage with historical and cultural knowledge that honors Black STEM narratives while providing curricular opportunities to foster liberatory imaginations that validate their STEM contributions.

Promoting a culturally relevant and engaging learning experience through the lens of Afrofuturism reveals the alternative organizational coalition’s use of educational curricula as mechanisms of gatebreaking systems and structures of access, and the overall significance for elevating Black ideologies in the biological sciences. The remembrance of Black histories involves disrupting historical erasure and weaponized amnesia, engaging the themes of *Reckoning with Black Marginalization* and *Reclaiming Black Legacies*. As structures in STEM higher education remain rooted in the institutionalization of anti-Blackness, it is imperative to refute structural gatekeeping that perpetuates deficit ideologies across biology and STEM contexts (Morton, 2023). Scholars have emphasized how gatekeeping continues to manifest through the prevalence of weed-out culture (Morton et al., 2019; Marshall et al., 2021; Weston et al., 2019; Wilkins-Yel et al., 2019). As such, gatekeeping emerges through curricular erasure and historical neglect. Gatebreaking comes from re-envisioning curriculum to meaningfully and intentionally account for historical and contemporary Black ontologies and STEM innovations.

We purposefully invoke stories of Black women, as detailed in the theme of *Reckoning with Black Marginalization,* to align with the Afrofuturist principle of feminine identity (Williams, 2026) and to exemplify the impact that Black women have had on the field of biology and, more importantly, on Black life. The history of medical exploitation is evident in the medical innovations developed from Lacks’ immortal cells (Khan, 2011). The non-consensual medical exploitation and commodification of Black individuals strips them of their humanity and agency. In addition, Henrietta Lacks’s legacy of revolutionizing the medical industry is indicative of Afrofuturist conceptualizations of the healing properties within Black feminine identities, connecting the intersections between Blackness, womanhood, strength, and representation (Strait and Conwill, 2023; Womack, 2013). Her HeLa cells provided a foundation for a variety of medical discoveries, including chemotherapy, *in vitro* fertilization, gene mapping, anti-cancer drugs, vaccine developments, and insights into the side effects of radiation (Khan, 2011; Klitzman, 2022).

Afrofuturist pedagogical practices and methods of instruction draw on poetics, poetry, dance, and play to envision a future in which Black contributions are celebrated (Boyd Acuff, 2020; Coles et al., 2026; Holbert et al., 2020). As detailed in *Re-envisioning Black Possibilities*, instructors can build on this framing by assigning performance tasks that intentionally push students to connect to Black legacies through 3-D art installations, storyboarding, posters, comic strips, and performance pieces. Students’ understandings of complex biological concepts (e.g., the disproportionate effects of cancer) can be explored and documented through their interactions with respective elders and family members, as highlighted by the theme of *Reclaiming Black Legacies*. The construction of student artifacts, developed through storyboarding, was designed to scaffold students’ understanding of family histories and experiences with cancer, and to analyze the extent to which students can create new realities free of cancer, thereby embodying the theme of *Re-envisioning Black Possibilitie*s. These pedagogical possibilities highlight how assessment practices that reject traditionally ascribed knowledge hierarchies can bridge multiplicative ways of knowing and being throughout STEM contexts as another example of Gatebreaking strategies utilized during the reimagining process.

By incorporating speculative imagination and situating biological content within structural contexts, the curriculum creates opportunities to foster critical reasoning, socio-political awareness, and creative problem-solving in students. In correlating the subject of DNA with different hairstyles, students’ understandings can be challenged to reflect on the intersectional nature of being a Black woman as they solidify DNA’s role in all living organisms. The above example represents a practical curricular model that positions the spectrum of Blackness as an inherent component of STEM ecosystems. While actively resisting the cultures and norms of disengagement and devaluation that often precede Black students’ entry into institutional and interpersonal contexts (McGee, 2016; McGee, 2020).

## Considerations

The current study has several considerations that readers must account for when interpreting its outcomes. First, the adaptations to the lesson plans and other curriculum documents should be used in future research focused on student engagement, identity development, and persistence in the biological sciences. The National Science Foundation’s cancellation of the REC-Network’s federal grant in 2025 is a structural barrier that our curriculum was designed to help students reckon with. Cutting these funds directly impacted the dissemination of our curriculum as it grapples with and acknowledges the historical harms that were done to Black people. The U.S. has seen a trend of defunding race-centered research, with efforts to dismantle Diversity, Equity, and Inclusion initiatives at both the federal and institutional levels (Consortium of Social Science Associations, 2025; National Science Foundation, 2025). Therefore, advocates and policymakers must understand that the current political climate is the documented reason for defunding our research; however, it is also the justification for the necessity of our curriculum.

## Implications and conclusion

In future studies, researchers should examine the experiences of Black undergraduate students who have participated in Afrofuturist biology lessons within an inclusive undergraduate STEM environment to determine how they develop long-term participation in science, identities, professional pathways, and community advocacy. This research focus shifts from curriculum analysis to the examination of Gatebreaking pedagogy and its impact on Black students’ STEM trajectories, giving insight into the content, procedural, conditional, and somatic knowledge acquired through an Afrofuturist biology course. As well, curriculum writers and practitioners, our findings provide a roadmap for designing culturally transformative curricula that center Black students’ identities, histories, and imaginaries. The REC-Network’s framework can extend beyond biology courses, as our Gatebreaking educational strategies are transferable to other STEM disciplines and can transform higher education and STEM environments broadly.

Overall, this research study aimed to illuminate how leveraging Afrofuturism to re-envision biology content, informed by Black knowledge systems and stories, functions as a Gatebreaking praxis directly countering exclusionary barriers historically enforced through gatekeeping practices in STEM education. Our study reveals the possible implications for using Afrofuturism as a conceptual framework to re-envision undergraduate STEM education through a speculative, African ancestral model that situates scientific inquiry and knowledge production. It also provides insight into what gatebreaking in biology education can look like as a curricular endeavor. Policymakers must recognize that anti-diversity, equity, and inclusion policies, as well as the political climate, have heightened the urgency of this study. Rescinding federal research funds does not increase the rigor of STEM; instead, it distorts the integrity of STEM disciplines by intentionally ignoring whose knowledge, histories, and futures STEM disciplines have always served. Therefore, the REC Network’s curriculum, presence, and emerging research draw on the Sankofa bird, which looks back to move forward. They emphasize that, for systematic gatebreaking to occur in STEM, Black students in the biological sciences deserve to learn with their histories in mind and to use their imagination freely to solve community issues.

## Data Availability

The datasets presented in this article are not readily available because IRB Approved Restrictions to Data Sharing. Requests to access the datasets should be directed to Terrell Morton: mortontr@uic.edu.
