# Supplementary material for: Reclaiming legacy and envisioning futures in STEM education: Afrofuturism and a scholar-activist-community coalition
Source: Front Sociol. 2026 Oct 14;11:1907915. doi: 10.3389/fsoc.2026.1907915 (PMC13713346; doi:10.3389/fsoc.2026.1907915)
Supplement: Supplementary file 1 [file Data_Sheet_1.pdf]

Guiding Questions: Enhancing Biology Lessons
Re-Envisioning Culture Network

An Afrofuturism Approach
Honoring Our Ancestors & Establishing Our Legacy

What is Afrofuturism?

- Engage with the interactive image to learn more about Afrofuturism.

Guiding Questions to consider as you reimagine your lesson.

- How can your lesson be adapted to be more futuristic & creative, student-centered, or include community projects?
- How are the ancestors honored in your lessons?
- How might developed solutions be presented with a Black Cultural technology-enhanced or utopian lens?

Topic, Learning Objectives, & Content:

- What biology content are you teaching?
  - Metabolism, Cancer, Anatomy & Melanin
- What topics in the Black community can be connected to Biology content?
  - Health Disparities, Mental Health Access, Food Deserts, Black Joy
- What Black scientists can be highlighted that will connect biology content and the Black community?
- How do your learning objectives encourage students to engage in inquiry and creative futuristic solution-making?

Afrofuturistic Adaptation of **Learning Activities and Tasks**: (10-15mins)

- Radical Future Ideation
  - Without current constraints, what would be the solutions?
- Future Shaping
  - Identify an aspect of your topic and consider how it has changed over time.
  - Name the current state of the topic.
  - How has it changed from the past?
  - What small changes led to the changes?
  - What could cause a radical change for the better?
- Mindmapping
  - Topic/idea/question in the middle of the page
  - Add branches from the topic to aspects/components of the main topic.
    - write on each branch how the component is connected to the main topic.
  - Could any of the smaller branches lead to an answer to the main topic?
- Storyboarding
  - Consider your topic; how can it be reimagined as a story?
  - How will people use your topic in the future?
  - Sketch your ideas in a storyboard template.
    - Don't forget to give your character(s) a voice.
    - What will your solution look like?
- Paper Prototyping
  - allows you to represent your ideas quickly and show how someone might interact with your ideas.
  - Using paper, create a visual model of your idea in the future.
    - Fold, cut, and draw to help convey your ideas.

Afrofuturistic Adaptation of **Assessment** Structure:

- Digital Solutions (online games, virtual reality, social media)
  - What have digital solutions looked like in the past (10, 25, 50 years ago)?
  - What do digital solutions look like today?
  - What might digital solutions look like in the future?
- Art Installations (art for social change, 3-D works)
  - How might solutions to your topic be displayed as art?
  - What will art look like in the future (10, 25, or 50 years)?
- Performance Arts (any art that is performed for a live audience)
  - poetry, dance, plays
  - What might the performances look like in 10, 25, or 50 years?
- Mutual Aid (an exchange of skills and resources to help a community)
  - How can the solutions you establish with the community prevent shortages in the future?

Bridge to Liberation – What specific resources and materials do we have access to, and what resources and materials will we need for Afrofuturistic and liberated adaptations (e.g., community partners, texts, images, technologies, etc.) What does liberation look like, sound like, feel like, smell like, or taste like to you?<br>● Community Partners<br>○ How will you connect and share space with community leaders/partners?<br>○ What can help your community feel encouraged, recognized, and thrive?<br>○ What makes you feel important, valued, and free?<br>○ How can you work with the community to preserve those feelings?<br>● Documentation (websites, archives, journals)<br>○ How are we documenting the present to be used for the future?<br>○ What documentation can we use from the past?<br>■ Texts<br>● What text can used to help students feel supported and seen?<br>■ Images<br>● What images can be used to challenge the dominant narrative of who we are as a Black community?<br>● Technologies<br>○ What technology exists that can help shape a person's experiences?<br>■ Think small (smart watches) to large (Google Nest: connect other devices)<br>○ How can storytelling be used as a technology to share cultural knowledge?
Additional Resources<br>● Assemble STEM Program (website)<br>● Building Utopia: Radical Futuring (slide deck)<br>● Chicago Community Gardens (website)<br>● Student-Centered Learning: It starts with the Teacher (website)<br>● Stanford Cancer Institute - Community Education & Science Café (website)

Adapted from the Building Utopia Deck https://www.buildingutopiadeck.com/

Glossary

A

- **Afrofuturism** is a way of imagining possible futures through a Black cultural lens. (Building Uptopia)
- **Art Installations:** three-dimensional works designed to transform spaces.

B

- **Black joy** is a racialized emotion that encompasses feelings of happiness, peace, wellness, healing, gratitude, and beauty (Muhammad, 2023; Tichavakunda, 2023). It is felt and shared among a collective of Black identities originating from the African Diaspora. As a tool, black joy serves as acts of refusal and resistance (Lewis-Giggetts, T.M., 2020), despite the adversity, dehumanization, and suffering that Blackness has historically been defined as (McKittrick, 2014).

C

- **Community projects** consider the needs of the community. Some projects might include community gardens, art installations, or building projects, all of which involve people working together and supporting one another.

D

- **Digital Solutions** can come from digital tools and platforms that offer electronic solutions and can extend the physical world. For example, virtual reality, social media, and even online games create a digital landscape full of possibilities.
- **Documentation** is a tool for record keeping, which includes websites, archives, journals, and zines. They can take place at any point in time and assist in remembering the past, and can help build the future.

E

F

- **Future Shaping** is a method for defining problems. Consider how a topic has changed dramatically over time. Consider what small changes led up to the larger change. What might cause a radical change for the better?

G

H

- **Honoring Our Ancestors** by looking to the past is a constant reflection process. This past may look different in different times, but the past, present, and future are linked by ancestry.

I

J

K

L

- **Liberation** happens when you reimagine your future without the systemic barriers threaded within societal institutions.

M

- **Mind mapping** is a method for defining problems and exploring opportunities for designs. Add your topic to the center of the page and connect branches with themes and ideas that connect to the next smaller topic.
- **Mutual Aid** is a way to exchange skills for resources and can also be considered bartering with others to meet community needs.

N

O

P

- **Paper Prototyping** is a method for testing ideas. It allows you to quickly represent how your ideas function with moving parts. For example, you could use pieces of paper to cut, fold, and draw to create a visual model.
- **Performance Arts** cover many art forms, including live poetry, dance, and any artistic performances presented live to an audience.

Q

R

- **Radical Future Ideation** is a planning method to define problems or opportunities. For instance, you could brainstorm solutions without the limits of today's society to current issues.

S

- **Storyboarding** is a method for ideation. It contains rough sketches and simple sentences explaining the sketch. For example, a product designer might use a storyboard to string together user stories and various research findings to develop technology requirements.
- **Student-Centered** is a teaching practice that places emphasis on students having an active role in why, what, and how their learning experiences are structured.

T

U

V

W

X

Y

Z
