# Supplementary material for: Reclaiming legacy and envisioning futures in STEM education: Afrofuturism and a scholar-activist-community coalition
Source: Front Sociol. 2026 Oct 14;11:1907915. doi: 10.3389/fsoc.2026.1907915 (PMC13713346; doi:10.3389/fsoc.2026.1907915)
Supplement: Supplementary file 2 [file Data_Sheet_2.pdf]

Tips for Developing Afrofuturistic-Oriented Instructional Activities

Re-Envisioning Culture Network

Considerations:

1. Afrofuturism challenges us to simultaneously engage past, present, and future within our approach.
2. Afrofuturism centers Black cultures, Black creativity, and Black possibilities expressed through the integration of art, music, science fiction (imagination), innovation (e.g., STEM), and liberatory praxis.
3. Afrofuturism pushes us to freedom dream in ways that are not bound by existing structures and limitations.

Tips:

1. Make activities engaging, allowing for dialogue, interactions, and shared problem-solving among participants.

2. Consider the power dynamics. Develop processes that account for power and privilege that come with demographic identities (e.g., race, gender, sexuality, religion, etc.) and group responsibilities (e.g., leader, note-taker, etc.) when establishing groups for group work.
    a. You want to ensure that people are not discriminated against because of their identities within groups.

3. Create activities that allow people to bring their cultural identities and perspectives into the learning space. This can be done through prompts or projects that allow for facts, processes, and conditions of a topic to be explored through a self-selected project.
