# Supplementary material for: Reclaiming legacy and envisioning futures in STEM education: Afrofuturism and a scholar-activist-community coalition
Source: Front Sociol. 2026 Oct 14;11:1907915. doi: 10.3389/fsoc.2026.1907915 (PMC13713346; doi:10.3389/fsoc.2026.1907915)
Supplement: Supplementary file 3 [file Data_Sheet_3.pdf]

Tips for Developing Afrofuturistic-Oriented Assessments
Re-Envisioning Culture Network

Background: There are two types of assessments to consider implementing within learning spaces, **formative assessments** and **summative assessments**.

- Formative: "in the moment" assessments implemented before, during, or immediately after instruction as a way to measure a learner's understanding of the ideas. Allows for real-time updates to and shifts in pedagogical practices to better account for students' needs and insights.
- Summative: An assessment that occurs at the end of a specific block of instruction (or all instruction) to determine the learner's final, overall understanding and achievement.

Considerations:

1. Afrofuturism challenges us to simultaneously engage past, present, and future within our approach.
2. Afrofuturism centers Black cultures, Black creativity, and Black possibilities expressed through the integration of art, music, science fiction (imagination), innovation (e.g., STEM), and liberatory praxis.
3. Afrofuturism pushes us to freedom dream in ways that are not bound by existing structures and limitations.

Tips:

1. Assessment should be reliable, valid, and bias-free.
    a. Reliable: Consistent with what it measures across contexts and groups.
    b. Valid: Supported by evidence.
    c. Bias-Free: Do not assume that everyone "knows" the same examples, latent messages, and symbolic meanings associated with words and ideas.

2. Be transparent about what you are intending to evaluate.
    a. The clearer and direct you are in what you are asking people to think through and consider, the better.

3. Provide opportunities to demonstrate learning that has occurred across the designated time period as opposed to at one time period, recognizing that everyone has a different starting point.
    a. Measuring at one time period evaluates what a person knows at one specific moment. Measuring across a time period evaluates what a person gained as a result of where they were before the ideas were presented on that specific topic.

4. Consider measuring declarative, procedural, conditional, and somatic knowledge associated with the concepts.
    a. Declarative: facts.
    b. Procedural: processes and approaches.
    c. Conditional: the conditions by which one applies the facts and specific processes.
    d. Somatic: embodied knowledge, what is felt, understood, and performed.

5. Incorporate mechanisms that allow the learner to bring in their identities and cultures within the evaluation process.
    a. Assessing core competencies associated with the ideas does not mean that you cannot create questions that allow students to bring themselves into it. Opportunities to "choose your own adventure" can allow students to bring in their identities and cultures while also demonstrating what they know about the topics explored.
